# Shared genetic loci for body fat storage and adipocyte lipolysis in humans

**DOI:** 10.1038/s41598-022-07291-4

**Published:** 2022-03-07

**Authors:** Agné Kulyté, Veroniqa Lundbäck, Peter Arner, Rona J. Strawbridge, Ingrid Dahlman

**Affiliations:** 1 Lipid Laboratory, Endocrinology Unit, Department of Medicine Huddinge, Karolinska Institutet, Huddinge, Sweden; 2 Institute of Health and Wellbeing, University of Glasgow, Glasgow, UK; 3 Cardiovascular Medicine Unit, Department of Medicine Solna, Karolinska Institutet, Stockholm, Sweden

**Keywords:** Endocrinology, Endocrine system and metabolic diseases, Diabetes, Obesity

## Abstract

Total body fat and central fat distribution are heritable traits and well-established predictors of adverse metabolic outcomes. Lipolysis is the process responsible for the hydrolysis of triacylglycerols stored in adipocytes. To increase our understanding of the genetic regulation of body fat distribution and total body fat, we set out to determine if genetic variants associated with body mass index (BMI) or waist-hip-ratio adjusted for BMI (WHRadjBMI) in genome-wide association studies (GWAS) mediate their effect by influencing adipocyte lipolysis. We utilized data from the recent GWAS of spontaneous and isoprenaline-stimulated lipolysis in the unique GENetics of Adipocyte Lipolysis (GENiAL) cohort. GENiAL consists of 939 participants who have undergone abdominal subcutaneous adipose biopsy for the determination of spontaneous and isoprenaline-stimulated lipolysis in adipocytes. We report 11 BMI and 15 WHRadjBMI loci with SNPs displaying nominal association with lipolysis and allele-dependent gene expression in adipose tissue according to in silico analysis. Functional evaluation of candidate genes in these loci by small interfering RNAs (siRNA)-mediated knock-down in adipose-derived stem cells identified *ZNF436* and *NUP85* as intrinsic regulators of lipolysis consistent with the associations observed in the clinical cohorts. Furthermore, candidate genes in another BMI-locus (*STX17*) and two more WHRadjBMI loci (*NID2, GGA3, GRB2*) control lipolysis alone, or in conjunction with lipid storage, and may hereby be involved in genetic control of body fat. The findings expand our understanding of how genetic variants mediate their impact on the complex traits of fat storage and distribution.

## Introduction

Obesity, with abdominal fat deposition, is a risk factor for common metabolic diseases. Both overall obesity, as assessed by body mass index (BMI), and a central fat deposition, as assessed by waist-hip ratio adjusted for BMI (WHRadjBMI), display strong genetic predisposition^1,2^. By genome-wide association studies (GWAS) numerous genetic risk loci for BMI and body fat distribution have been identified^1,2^. In most cases, the underlying susceptibility genes in these loci and regulatory pathways remain to be defined. Although brain regulation of food intake is believed to be of central importance for BMI, local regulation of metabolism in peripheral organs may also be important^3^. In addition, expression of genes in GWAS loci associated with WHRadjBMI is enriched in adipose tissue, and some are differentially expressed between different adipose depots, giving support to the hypothesis that genetic susceptibility to high WHRadjBMI is mediated by effects on local metabolic pathways in adipose tissue^4^.

One mechanism in adipose tissue implicated in the control of fat mass is hydrolysis of triglycerides, i.e., lipolysis. High spontaneous and low catecholamine-stimulated lipolysis are associated with higher body weight in both cross-sectional and prospective cohorts^5,6^. Disturbances in adipocyte lipolysis have also been associated with central fat deposition^4,7^. We have recently reported the first GWAS of abdominal spontaneous and isoprenaline-stimulated adipose lipolysis in the unique GENiAL cohort^8^.

The present study aimed to determine whether genetic variants identified by GWAS to be associated with BMI^9^ and/or central fat deposition (e.g. WHRadjBMI)^10^ overlap with genetic variants reported to be associated with lipolysis^8^. To link genetic variants to specific genes, overlapping risk alleles for these traits were thereafter examined for allele-dependent gene expression through in silico analysis. Genes with allele-dependent expression patterns in adipose tissue were functionally evaluated in human adipose derived mesenchymal stem cells (hASCs)*.*

## Materials and methods

### Participants

The GENiAL cohort and GWAS of abdominal subcutaneous adipose tissue (SAT) lipolysis have been described previously^8^. WHRadjBMI was calculated in a sex-specific manner by inverse-normal transformation of the residuals of the linear regression model: WHR adjusted for age, age^2^ and BMI^11^. Measures of adipocyte lipolysis were available in a total of 939 study subjects (687 women, 252 men). Fifty-seven percent were obese (defined as BMI ≥ 30 kg/m^2^). 194 participants had type 2 diabetes, hypertension, or dyslipidemia alone or in different combinations.

### Ethics

The study was approved by Forskningsetikkommittén vid Huddinge universitetssjukhus, that is the local committee on research ethics at Huddinge hospital (Sweden) (reference number 167/02) and all methods were performed in accordance with relevant guidelines and regulations. The study wa**s** explained in detail to each participant and informed consent was obtained; this was in written form since 1996 when it became obligatory.

### Adipocyte lipolysis measurement in GENiAL

Adipocyte lipolysis in clinical samples were measured as previously described^8^. Briefly, abdominal SAT biopsies obtained by needle aspiration were subjected to collagenase treatment to obtain isolated adipocytes. Fat cell suspensions (diluted to 2% volume/volume) were incubated for 2 h at 37 °C with air as the gas phase in Krebs–Ringer phosphate buffer (pH 7.4) supplemented with glucose (8.6 mmol/l), ascorbic acid (0.1 mg/ml), and bovine serum albumin (20 mg/ml) with the synthetic non-selective β-adrenoreceptor agonist isoprenaline (Hässle, Mölndal, Sweden) at concentrations (10^−9^–10^−5^ mol/l; stimulated lipolysis) or without (spontaneous lipolysis). Quantity of glycerol as a measure of lipolysis was evaluated in an aliquot of medium at the end of the incubation^12^. Spontaneous lipolysis was expressed as glycerol release/cell weight multiplied by the estimated abdominal subcutaneous fat mass, i.e., an estimate of the total release of glycerol from the area which is the site for the biopsy^8^. Herein we expressed isoprenaline-stimulated lipolysis as the quotient of glycerol release at the maximum effective isoprenaline concentration divided by the spontaneous rate (no hormone present) of glycerol release from the isolated fat cells. Values were log_10_ transformed to improve normality.

### Genetic analysis

As previously described, the genetic analysis of spontaneous and stimulated lipolysis in GENiAL was performed in PLINK using linear regression, assuming an additive genetic model and including age, sex and BMI as covariates^8^. After quality control, 9,260,588 SNPs were analysed in 886 study subjects^8^.

### Data mining

In our analysis of BMI we used the SNPs listed in the file Meta-analysis_Locke_et_al + UKBiobank_2018_top_941_from_COJO_analysis_UPDATED which was reached by a link in Yengo et al.^2^ to https://portals.broadinstitute.org/collaboration/giant/index.php/GIANT_consortium_data_files and 2.2 BMI and Height GIANT and UK BioBank Meta-analysis Summary Statistics.

In our analysis of WHRadjBMI we used the SNPs listed in the file whradjbmi.giant-ukbb.meta.1.merged.indexSnps.combined.parsed which was reached by a link from Supplementary Table 1 in Pulit et al.^1^ to https://github.com/lindgrengroup/fatdistnGWAS.

The FANTOM 5 dataset^13^ was used to assess gene expression in human adipose derived stem cells (hASCs) undergoing in vitro differentiation to adipocytes. Transcripts whose expression were > 10 tags per million (TPM) at some point during the time course were defined as detected in hASCs.

Candidate genes were selected based on expression quantitative trait locus (eQTL) data in the GTEx^14^ (www.gtexportal.org) database and were retrieved in December 2019 and August 2020. All SNP-gene pairs listed in GTEx and reported herein reach FDR < 5%. p-values for eQTL genes are nominal and taken from GTEx. There was no threshold for physical distance. Among SNP-gene pairs in Tables 1 and 2, four genes were located > 100 Kb from the SNP; only one gene was located at a distance > 500 Mb from the SNP, *EBPL* (~ 1 Mb distance).

**Table 1:** eQTL SNPs displaying congruent association with adipocyte lipolysis and BMI.

SNP	Chrom	Pos	Spontaneous lipolysis					BMI					eQTL (SAT)				Detected in hASCs
			Beta	*p*	Ref	Alt	Freq	Beta	*p*	Ref	Alt	Freq	Gene	*p*	High	Low	
rs967605	1	23399932	0.07	7.78E−03	T	C	0.14	− 0.02	3.68E−13	C	T	0.83	*ZNF436*	0.0000086	C	T	Yes^a^
													*ZNF436-AS1*	0.000018	C	T	n.d
rs10923724	1	119546842	0.05	8.33E−03	T	C	0.40	− 0.01	1.14E−12	C	T	0.57	*WARS2*	1.70E−46	C	T	Yes^a^
													*TBX15*	0.0000097	T	C	Yes^a^
rs919433	2	198166565	− 0.04	2.67E−02	A	G	0.62	0.01	1.36E−11	G	A	0.36	*ANKRD44*	7.30E−13	G	A	n.d
rs4515655	9	128616073	− 0.04	4.37E−02	C	T	0.38	− 0.01	1.01E−09	C	T	0.40	*PBX3*	3.10E−17	T	C	n.d
rs17105272	14	77529783	− 0.04	4.15E−02	T	C	0.67	0.01	2.22E−09	C	T	0.32	*RP11-7F17.3*	4.20E−09	T	C	n.d

SNP	Chrom	Pos	Isoprenaline stimulated lipolysis					BMI					eQTL (SAT)				Detected in hASCs
			Beta	*p*	Ref	Alt	Freq	Beta	*p*	Ref	Alt	Freq	GENE	*p*	High	Low	
rs967605	1	23399932	− 0.06	1.63E−03	T	C	0.14	− 0.02	3.68E−13	C	T	0.83	*ZNF436*	8.60E−06	C	T	Yes^a^
													*ZNF436-AS1*	1.80E−05	C	T	n.d
rs2850969	4	102183594	0.04	2.81E−02	C	T	0.85	− 0.01	2.16E−09	C	T	0.86	*FLJ20021*	1.90E−11	T	C	Yes^a^
rs450231	9	101481205	− 0.03	2.76E−02	A	G	0.29	− 0.01	2.59E−12	G	A	0.75	*ANKS6*	0.000065	G	A	n.d
rs10118701	9	103061366	0.04	1.02E−02	G	A	0.66	− 0.01	3.46E−12	G	A	0.68	*INVS*	1.70E−13	G	A	Yes^a^
													*STX17*	1.50E−07	G	A	Yes^a^
rs4515655	9	128616073	0.03	4.34E−02	C	T	0.38	− 0.01	1.01E−09	C	T	0.40	*PBX3*	3.10E−17	T	C	n.d
rs3781099	10	27318776	0.05	4.67E−02	T	C	0.92	0.02	8.07E−11	C	T	0.08	*MASTL*	4.20E−11	C	T	n.d
													*LINC00202-1*	0.0000022	T	C	n.d
rs1558236	12	111780998	− 0.09	2.09E−02	G	C	0.97	0.06	9.85E−15	G	C	0.97	*ALDH2*	0.0000018	G (rare)	C	Yes^a^
rs2537847	17	65694355	0.03	3.14E−02	G	A	0.25	− 0.01	3.39E−09	G	A	0.24	*RP11-855A2.1*	0.000071	A	G	n.d

Where *Chrom.* chromosome, *Pos.* genome position in basepairs according to GRCh37, Beta refer to Alt allele, *Freq* frequency of Alt allele, *High* allele with highest gene expression according to GTEx; n.d. = mRNA not detected in hASC undergoing in vitro differentiation to adipocytes.

^a^Expression is presented in the Supplementary Fig. 1.

**Table 2:** eQTL SNPs associated with adipocyte lipolysis and WHRadjBMI.

SNP	Chrom	Pos	Spontaneous lipolysis					WHRadjBMI				eQTL (SAT)			Detected in hASCs
			Beta	*p*	Ref	Alt	Freq	Beta	Ref	Alt	Freq	Gene	High	Low	
rs10923724	1	119546842	0.05	8.33E−03	T	C	0.40	0.03	C	T	0.56	*WARS2*	C	T	Yes^b^
												*TBX15*	T	C	Yes^b^
rs10055995	5	137698299	− 0.06	5.17E−04	T	C	0.42	− 0.01	C	T	0.58	*REEP2*	C	T	n.d
												*KDM3B*	T	C	n.d
rs10980797	9	113912553	0.04	1.75E−02	G	A	0.51	− 0.02	G	A	0.52	*LPAR1*	A	G	n.d
rs747249	11	130271647	0.04	2.74E−02	G	A	0.35	0.01	G	A	0.36	*ADAMTS8*^a^	G	A	n.d
rs10878367	12	66436097	− 0.05	2.04E−02	A	T	0.73	0.02	T	A	0.30	*LLPH-AS1*	A	T	n.d
rs12325187	16	3364997	− 0.04	2.27E−02	G	C	0.69	0.01	G	C	0.73	*TIGD7*	G	C	n.d
												*ZNF263*	G	C	Yes^b^
												*LINC00921*	C	G	n.d
												*ZNF200*	G	C	n.d
												*MTCO1P28*^a^	G	C	n.d
rs4794033	17	47358481	−0.06	1.68E−02	A	G	0.88	− 0.02	G	A	0.10	*ZNF652*	A	G	n.d
rs1328757	20	56135199	0.05	4.73E−03	T	C	0.56	0.01	C	T	0.47	*PCK1*	T	C	Yes^b^

SNP	Chrom	Pos	Isoprenaline simulated lipolysis					WHRadjBMI				eQTL (SAT)			Detected in hASCs
			Beta	*p*	Ref	Alt	Freq	Beta	Ref	Alt	Freq	Gene	High	Low	
rs10055995	5	137698299	0.03	1.03E−02	T	C	0.418	− 0.01	C	T	0.58	*REEP2*	C	T	n.d
												*KDM3B*	T	C	n.d
rs13198178	6	41702227	− 0.05	4.30E−02	C	G	0.933	0.03	G	C	0.06	*TFEB*	G	C	n.d
rs10878367	12	66436097	0.03	2.64E−02	A	T	0.727	0.02	T	A	0.30	*LLPH-AS1*	A	T	n.d
rs797486	13	51221618	0.05	1.04E−02	A	C	0.153	0.04	C	A	0.89	*EBPL*	A	C	Yes^b^
rs146182298	14	52531408	− 0.09	2.47E−02	T	C	0.969	0.03	C	T	0.04	*NID2*^a^	C	T	Yes^b^
rs11074934	16	10979440	0.04	1.15E−02	T	C	0.737	0.01	C	T	0.27	*CIITA*	T	C	n.d
rs4794033	17	47358481	0.05	2.08E−02	A	G	0.883	− 0.02	G	A	0.10	*ZNF652*	A	G	n.d
rs9909443	17	73308346	− 0.04	3.85E−03	T	C	0.780	− 0.01	C	T	0.23	*GGA3*	T	C	Yes^b^
												*MRPS7*	C	T	Yes^b^
												*NUP85*	T	C	Yes^b^
												*MIF4GD*	C	T	n.d
												*GRB2*	T	C	Yes^b^
rs4646342	17	17493272	− 0.03	3.91E−02	A	G	0.550	− 0.02	G	A	0.45	*PEMT*	G	A	Yes^b^
rs8126001	20	62711459	0.03	2.98E−02	T	C	0.516	− 0.02	C	T	0.49	*OPRL1*	T	C	n.d
												*RGS19*	T	C	n.d

Where *Chrom*. chromosome, *Pos*. genome position in basepairs according to GRCh37, Beta refer to Alt allele, *Freq* frequency of Alt allele, *High* allele with highest gene expression according to GTEx; *n.d.* mRNA not detected in hASC undergoing in vitro differentiation to adipocytes.

^a^eQTL in visceral adipose tissue.

^b^Expression is presented in the Supplementary Fig. 1.

### Mendelian randomisation

Mendelian randomisation (MR) analysis was conducted using two sample MR as implemented by the online mrbase software (mrbase.org/pmid29846171). SNPs that were identified as being shared loci (GWAS-significant loci for BMI or WHRadjBMI and nominally significant (p < 0.05) for basal or stimulated lipolysis) were used as the exposure. For BMI, SNPs were also filtered for effect direction consistency. The largest datasets to date for BMI^2^ and WHRadjBMI^10^ were used as outcomes for this analysis. For BMI, the dataset used to select SNPs above was consistent with that used here^2^. For WHRadjBMI, the data set used above^1^ was not available in MRbase, therefore the largest GWAS of WHRadjBMI available was used instead^10^. Settings chosen were: No check for LD between SNPs, Minimum LD Rsq for proxies = 0.8, MAF threshold for palindromes = 0.3, attempt to align strands for palindromic SNPs (all default settings), analyses using MR Egger, MR Egger (bootstrap), Weighted median, Inverse variance-weighted, weighted mode and weighted mode (NOME). The possibility of horizontal pleiotropy and fulfilment of the NOME assumption was tested using the I2 statistic, calculated as per Bowden et al.^15^.

### Cell culture and transfection with small interfering RNA

Isolation, growth, differentiation, and characterization of hASCs isolated from subcutaneous adipose tissue of a male donor (16 years old, BMI 24 kg/m^2^) donor have been described in detail previously in Refs.^13,16^. hASCs were transfected 24 h before induction of adipogenesis using ON-TARGETplus SMARTpool small interfering RNAs (siRNAs) targeting candidate genes or control siRNA #1 (Dharmacon/Horizon Discovery Group, Lafayette, CO, US) and Dharmafect 3 transfection agent (Dharmacon/Horizon Discovery Group) as previously described^17^. Differentiation to adipocytes was started by medium replacement 24 h post-transfection and medium was subsequently replaced every third day. The RNA and medium were collected at days 2, 6 and 9 after the induction of differentiation and cells were fixed for lipid staining at day 9.

For *siNUP85* hASCs cells were also transfected 24 h postinduction of differentiation using a NEON electroporator (Invitrogen, Carlsbad, CA, US) according to the manufacturer's protocol. Briefly, for one transfection reaction 1 million hASCs suspended in 90 µl of R buffer was mixed with 200 pmol of siRNA targeting *NUP85* or a control siRNA #1 and transfection was performed using 100 µl NEON electroporation tip. Electroporation conditions were 1600 V, 20 ms width, 1 pulse. Subsequently, cells were plated in antibiotic free medium at a density of 120,000 cells/well in 24-well plates or 10,000 cells/well in 96-well plates. Medium was replaced 24 h post-transfection and subsequently every third day. For this type of experiment, the cells were cultured until day 3, 6 and 10 of differentiation, at which time RNA and medium were collected or cells were fixed for lipid staining.

Product identification numbers for siRNA are listed in Supplemental Table 1.

### Measurement of lipolysis and lipid accumulation in in vitro differentiated adipocytes

For glycerol measurements, medium was collected at day 9 of differentiation of hASCs for all tested genes and additionally at day 10 for si*NUP85* when transfected at day 1 of differentiation*.* Glycerol is a valid end-product of lipolysis as it is re-utilized by fat cells to a negligible extent and was measured in cell culture medium as described^18^. Amounts of glycerol were normalized to the number of cells (evaluated as stained nuclei and described below) in each well.

Assessment of isoprenaline-stimulated lipolysis in si*NUP85*-transfected hASCs was performed as described previously^19^. In brief, cells were incubated for 3 h in DMEM/F12 medium supplemented with 20 mg/ml of BSA and with or without isoprenaline (1 µmol/l). Glycerol in media was measured as described above. On collection of medium, cells from the same wells were lysed in RIPA buffer, protein concentration was measured, and glycerol levels were then corrected for total protein content.

Lipids were stained and quantified at differentiation day 9 for all the genes and additionally on day 10 for *siNUP85* as previously described^8^. Briefly, following fixation with paraformaldehyde neutral lipids were stained with Bodipy 493/503 (0.2 µg/ml; Molecular Probes, Thermo Fisher Scientific, Waltham, MA, US) and nuclei (DNA) were stained with Hoechst 33342 (2 µg/ml; Molecular Probes) for 20 min at room temperature. Accumulation of intracellular lipids (Bodipy) and cell number (Hoechst) were quantified with CellInsight CX5 High Content Screening (HCS) Platform (Thermo Fischer Scientific) with integrated protocols. Total Bodipy fluorescence (lipid droplets) was normalized to the amount of nuclei in each well.

### RNA isolation and analysis of gene expression

NucleoSpin RNA II kit (Macherey–Nagel, Düren, Germany) was used to extract total RNA from adipocytes. RNA concentration and quality were measured using Varioskan LUX multimode microplate reader and µDrop plate (ThermoFisher Scientific). Subsequently, 50 ng of total RNA was reverse transcribed using the iScript cDNA synthesis kit (Bio-Rad, Hercules, CA, US). Quantitative RT-PCR was performed using 2.5 ng of cDNA and commercial TaqMan assays (Applied Biosystems/Thermo Fisher Scientific) in 10 μL reaction on the CFX96 Touch qPCR Detection System (Bio-Rad) or QuantStudio Real-Time PCR (Thermo Fisher Scientific). Relative gene expression was calculated using the 2^−ΔCt^ method^20^. Taqman assays are listed in Supplementary Table 1.

### Microarray assay and analysis

Clariom S arrays (Thermo Fisher Scientific) were used for global transcriptome profiling of primary adipocyte cultures treated with siRNA against *ZNF436* (n = 6), *NUP85* (n = 6), or respective non-targeting siRNA control (n = 6) at day 6 of differentiation. The arrays were pre-processed in Transcriptome Analysis Console (Thermo Fisher Scientific) using SST-RMA, which includes normalization, summarization of probes to probesets, and background correction. We excluded 6,341 (*ZNF436* samples) and 6,316 (*NUP85* samples) probe sets with detection *p* > 0.05 in at least one sample i.e., not detected in at least one sample, leaving 15,107 and 15,132 probe sets, respectively, for downstream analysis.

SAMR using block permutations was used to detect differentially expressed genes^21^. Webgestalt (www.webgestalt.org) was used to identify differentially expressed gene ontologies between siRNA treated and control samples. The KEGG (https://www.genome.jp/kegg/) database was used to identify genes in the gluconeogenesis^22,23^, and wikipathways (https://www.wikipathways.org) in the fatty acid biosynthesis pathway.

### Statistical analysis

Data from in vitro experiments were analysed by t-test. All p-values are two-tailed. A binomial test in Excel was used to analyse whether there was evidence that proportion of BMI and WHRadjBMI SNPs showing a directionally consistent effect on lipolysis was larger than the number expected by chance, i.e., 50%. When specific hypotheses are tested, a nominal p < 0.05 is used as the significance threshold. In explorative transcriptome analysis multiple testing is taken into account and FDR 10% used as threshold. Correlation between lipolysis and thermogenic gene expression was evaluated by linear regression in JMP 14.

## Results

### Shared genetic loci for body fat storage and adipocyte lipolysis

**Figure 1:**
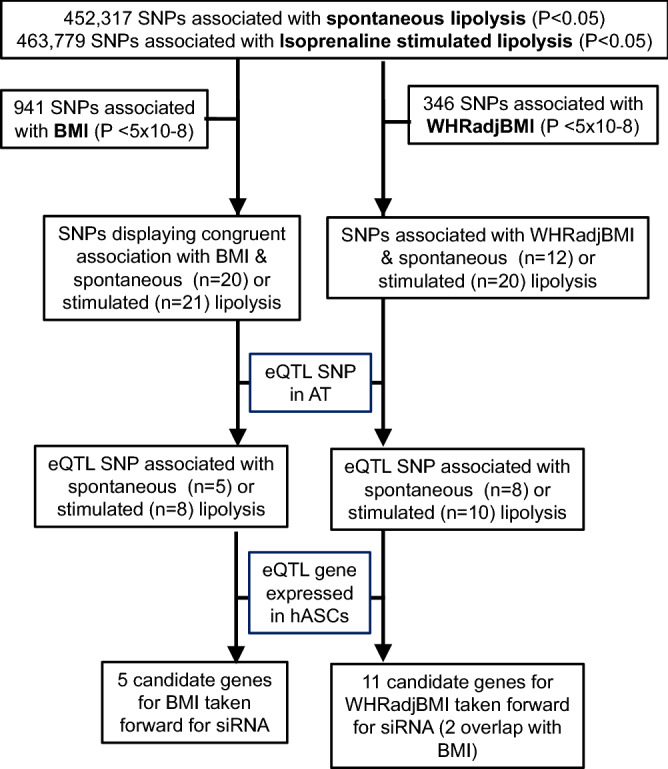
Flowchart describing the project.

Data mining to identify shared genetic loci for BMI and adipocyte lipolysis is summarized in Fig. 1. A lookup of SNPs previously associated with BMI^2^ was conducted in the results of the GWAS of lipolysis^8^. Among 941 SNPs reported to be associated with BMI^2^, 20 displayed nominal association (p < 0.05) with spontaneous lipolysis with consistent effect directions for BMI and spontaneous lipolysis (Supplementary Table 2). Five of these SNPs demonstrated expression quantitative trait loci (eQTLs) in SAT according to GTEx (Table 1). Furthermore, of 941 SNPs associated with BMI, 21 displayed nominal association with isoprenaline-stimulated lipolysis with expected discordant effect direction for BMI and stimulated lipolysis (Supplementary Table 3). Eight of the latter SNPs demonstrated eQTLs in SAT according to GTEx (Table 1).

We next assessed genetic influence on central fat distribution. Among index-SNPs in 346 genetic loci associated with WHRadjBMI^1^, 12 displayed nominal associations (*p* < 0.05) with spontaneous lipolysis; eight of these SNPs demonstrated eQTLs in SAT according to GTEx (Table 2, Supplementary 4). 20 SNPs displayed associations with WHRadjBMI and isoprenaline-stimulated lipolysis (Supplementary Table 5) of which ten SNPs were eQTLs in SAT (Table 2). In the case of WHRadjBMI, effect directions were not considered because there is limited information regarding the relationship between lipolysis in different adipose depots and adipose distribution. None of the SNPs mentioned herein are among SNPs previously reported in the GENiAL cohort to display GWAS suggestive association with lipolysis^8^.

### Functional evaluation of candidate genes in shared BMI-lipolysis loci

**Figure 2:**
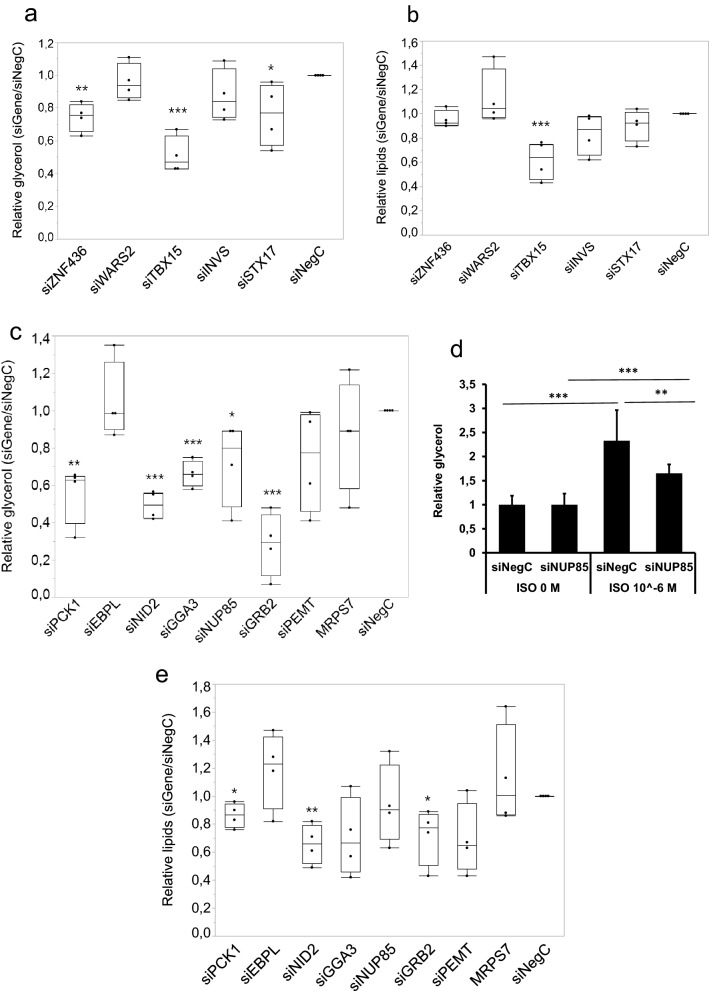
Effects of RNA interference-mediated knock-down on lipolysis and lipids in human adipose-derived stem cells. hASCs were transfected with control siRNA oligonucleotide (siNegC) or indicated siRNAs targeting specific genes. (**a**,**b**) Expression of *ZNF436*, *WARS2*, *TBX15*, *INVS* and *STX17* was knocked down using siRNA in hASCs 24 h prior induction of differentiation and followed until differentiation day 9, upon which accumulated glycerol in medium was measured to assess spontaneous lipolysis (**a**) and accumulation of neutral lipids was evaluated by lipid staining (**b**). (**c**,**e**) Expression of *PCK1*, *EBPL*, *NID2*, *GGA3*, *MRPS7*, *NUP85*, *GRB2* and *PEMT* was knocked down using siRNA in hASCs 24 h prior induction of differentiation and followed until differentiation day 9, upon which glycerol in medium was measured to assess spontaneous lipolysis (**c**) and accumulation of neutral lipids was evaluated in the cells (**e**). Results are based on four biological/independent experiments. Results were analyzed using t-test and presented in fold change ± SD relative to negative control Neg C. ***p < 0.005, **p < 0.01, *p < 0.05. (**d**) Expression of *NUP85* was knocked down using siRNA in hASCs 24 h before induction of adipogenenic differentiation of hASCs. At day 9 of differentiation cells were incubated with isoprenaline (ISO) and the effects of knock-down on lipolysis were evaluated. Results are based on four biological/independent experiments. Results were analyzed using t-test and are presented as fold change ± SD relative to 0 M isoprenaline (ISO) of respective condition (siNegC or siNUP85). ***p < 0.005, **p < 0.01.

Three eQTL genes (*ZNF436*, *WARS2*, *TBX15*) in the shared BMI-spontaneous lipolysis loci and five eQTL genes (*ZNF436*, *FLJ20021*, *INVS*, *STX17*, *ALDH2*) in the shared BMI-stimulated lipolysis loci were expressed in hASCs (Table 1, Supplementary Fig. 1a). Of these, five genes (*ZNF436*, *WARS2*, *TBX15*, *INVS* and *STX17*) were taken forward for functional siRNA screen in hASCs*. FLJ20021* was excluded as, until recently, it has been annotated as an uncharacterized lncRNA^24^. *ALDH2* was excluded because it has recently been established as a positive regulator of adipocyte differentiation^25^. We knocked-down the five candidate genes prior to induction of differentiation, followed by evaluation of glycerol levels in conditional medium as a marker of lipolysis. Knock-down of *ZNF436, TBX15* and *STX1*, but not *WARS2* and *INVS*, decreased glycerol levels relative to negative control siRNA (Fig. 2a). To assess whether the impact on lipolysis was direct or secondary to disturbances in adipogenesis, we stained the cells for neutral lipid content. Knock-down of *TBX15,* but not the other investigated genes, decreased amount of lipids (Fig. 2b). Results for the BMI-panel genes *ZNF436* and *STX17* were of particular interest because knock-down of these two genes affected glycerol release without affecting lipid content supporting the hypothesis that the impact on lipolysis was direct, and not indirect via influence on adipogenesis.

**Figure 3:**
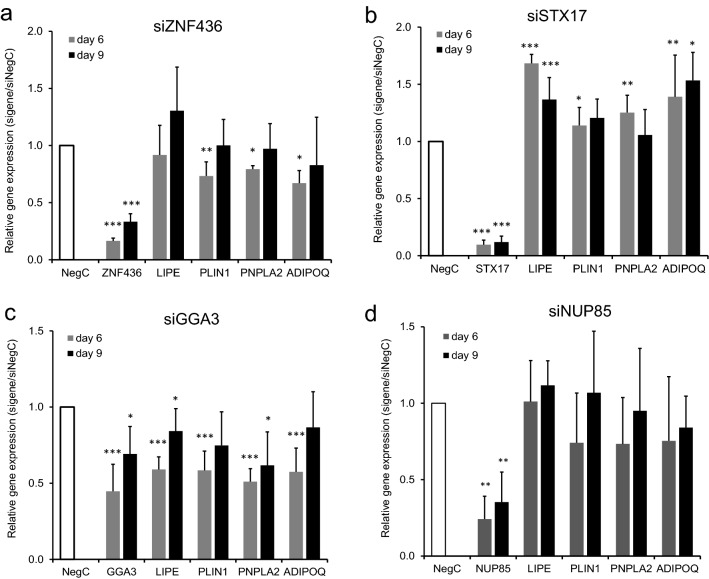
Effects of RNA interference-mediated knock-down of candidate genes in human human adipose-derived stem cells. Human adipose-derived stem cells were transfected with control siRNA (siNegC) or indicated siRNAs targeting specific genes (*ZNF436*, *STX17*, *GGA3* and *NUP85*) and followed until differentiation day 6 and 9, upon which the expression of target genes, *LIPE, PLIN1, PNPLA2* and *ADIPOQ* was monitored. Relative gene expression was normalized to the reference gene *18s*. Results were analyzed using t-test and presented as fold change ± SD relative to control siRNA (NegC) of corresponding time point (n = 6, for *GGA3* n = 11). ***p < 0.005, **p < 0.01, *p < 0.05.

In parallel, we evaluated the effect of knock-down efficiency of the studied genes at the mRNA level. In general, knock-down efficiency was good, i.e., varied between 90 and 70% with exception of *TBX15* where knock-down efficiency reached only 50% at day 6 (Fig. 3a, b and Supplementary Fig. 2a–c). Since for *TBX15* no intrinsic effect on lipolysis could be ensured the limited knockdown was considered to be of less importance. For the genes affecting glycerol we evaluated whether gene knock-down effects could be linked to altered expression levels of key genes such as *LIPE* and *PNPLA2* encoding lipases, *PLIN1* encoding the lipid coating protein Perilipin 1, or expression of *APIDOQ* encoding the adipocyte marker adiponectin. Knock-down of *ZNF436* caused temporary downregulation of *PLIN1, PNPLA2* and *ADIPOQ* (Fig. 3a). Knock-down of *STX17* upregulated *LIPE* and *ADIPOQ* at both day 6 and 9 of differentiation, while upregulation of *PLIN1* and *PNPLA2* was temporal (Fig. 3b). Knock-down of *TBX15* had minor effects on studied gene expression (Supplementary Fig. 2b).

### Functional evaluation of candidate genes in shared WHRadjBMI-lipolysis loci

We next selected eQTL genes in the shared WHRadjBMI-lipolysis loci for functional evaluation in vitro*.* Four genes in the shared WHRadjBMI-spontaneous lipolysis loci were expressed in hASCs: *TBX15*, *WARS2* (both included in analysis of BMI-loci above)*, ZNF263* and *PCK1* (Table 2). Seven genes in the shared WHRajdBMI-stimulated lipolysis loci were expressed in hASCs: *EBPL, NID2*, *GGA3, MRPS7, NUP85, GRB2*, and *PEMT* (Table 2, Supplementary Fig. 1b). Knock-down of *PCK1, NID2, GGA3, NUP85* and *GRB2* decreased levels of glycerol by 20–70% (Fig. 2c). Knock-down of *EBPL, MRPS7* and *PEMT* had no effect (Fig. 2c). Among the genes controlling glycerol release, accumulation of lipids was decreased by knock-down of *PCK1, NID2* and *GRB2.* (Fig. 2e). Knock down of *ZNF263* showed no effect on glycerol or lipid levels most likely due to due to poor knockdown in the tested conditions (data not shown).

The inhibition of spontaneous lipolysis by *PCK1* knock-down was not consistent with the genetic data, where the T allele is associated with high gene expression and low spontaneous lipolysis, and this gene was therefore irrelevant for further studies. *NUP85* and *GGA3* were the only two genes from the WHRadjBMI panel that affected glycerol release without affecting lipid content. However, knocking down *GGA3* at different time points during differentiation decreased lipid accumulation (data not shown). Based on this and its effect on key adipocyte genes (Fig. 3c, also see below) we could not rule out an effect of adipogenesis. Thus, *NUP85* was the only remaining gene from the WHRadjBMI panel that affected glycerol accumulation without affecting lipid content supporting that the impact on lipolysis was direct, and not indirect via influence on adipogenesis. Since the *NUP85* SNP rs9909443 was associated with isoprenaline-stimulated lipolysis (Table 2), we also evaluated effects of *NUP85* knock-down on isoprenaline-stimulated lipolysis, which was decreased compared to the control siRNA (Fig. 2d). In this experiment in basal condition (no isoprenaline) we did not detect lower glycerol release in si*NUP85* samples compared to control siRNA. However, this is only a 3 h “window” of lipolysis and the accumulative effect seems to be important to obtain effects on glycerol release by *NUP85*.

As for BMI genes, we evaluated knock-down efficiency of WHRadjBMI genes and assessed whether expression of known lipolysis genes could be affected. Knock-down efficiency varied from 75 to 95% (Supplementary Fig. 2e–i) except for *PCK1* and *GGA3* (Supplementary Fig. 2d and Fig. 3c). These genes were very low expressed in the beginning of differentiation and therefore it is possible that the siRNA-transfection conditions need to be further optimized. Furthermore, knock-down of WHRadjBMI-associated genes had less effect on adipocyte gene expression than BMI-associated genes. Knock-down of *PCK1* upregulated *LIPE* and temporally downregulated *PNPLA2* and *ADIPOQ* (Supplementary Fig. 2d). Knock-down of *GGA3, NID2* and *GRB2* temporary downregulated all studied genes (Fig. 3c and Supplementary Fig. 2f, h) while knock-down of *NUP85* had no effect (Fig. 3d).

### ZNF436 and NUP85 target genes

The analysis of expression of central genes controlling lipolysis (*LIPE*, *PLIN1*, and *PNPLA2*) did not reveal a functional link to lipolysis for the shared GWAS-candidate genes for BMI/WHRadjBMI and lipolysis. Therefore, two genes were taken forward for global transcriptome analysis: *ZNF436* from the BMI and *NUP85* from the WHRajdBMI analysis*.* They were selected because the genetic, metabolic, and functional results for lipolysis were consistent, e.g., rs967605-C was associated with high *ZNF436* expression, BMI and spontaneous lipolysis, and *ZNF436* knock-down inhibited glycerol release. Furthermore, for both genes it was reasonable to assume that the impact on lipolysis was indirect via influence in gene expression; *ZNF436* is a transcription factor^26^ and *NUP85* encodes a protein forming part of nuclear pores, which in turn are involved in transcriptional regulation^27–29^.

Transcriptome analysis revealed 396 genes that were differentially expressed between *ZNF436* knock-down and control cells with FDR < 10%, of which 203 were downregulated (Supplementary Table 6). Downregulated genes were enriched for the gene ontology (GO) “regulation of lipid metabolic process” Table 3). Expression of three genes (*PNPLA3*, *PDE3B* and *MGLL*) reported to be involved in lipolysis^30^ were among *ZNF436* regulated genes (Supplementary Table 6, Supplementary Fig. 3). There was no evidence of a compensatory impact on gluconeogenesis or fatty acid biosynthesis as only gene in each of these pathways were affected by *ZNF436* knockdown, i.e. *ENO1* (fold change 0.66) and ACSL4 (0.59).

**Table 3:** Gene sets overrepresented among ZNF436 or NUP85 regulated genes.

GeneSet	Description	Expected number of genes	Enrichment ratio	*p*	FDR	Gene symbols
**Gene sets overrepresented among genes downregulated by by *ZNF436* knockdown**						
GO:0062012	Regulation of small molecule metabolic process	3	4	3.49E−05	0.03	*FABP3;IGF1;SQLE;ENO1;IDI1;ABCD2;MVD;FDPS;GPER1;KAT2A;HMGCR;HMGCS1;STARD4*
GO:0008202	Steroid metabolic process	3	4	1.02E−04	0.04	*DHCR24;AGT;SQLE;IDI1;MVD;FDPS;ACAT2;HMGCR;CYP4V2;HMGCS1;STARD4*
GO:1901615	Organic hydroxy compound metabolic process	4	3	1.54E−04	0.04	*DHCR24;IGF1;SQLE;IDI1;PLCB2;MVD;FDPS;GPER1;ACAT2;HMGCR;CYP4V2;HMGCS1;PCBD1;STARD4*
GO:0035051	Cardiocyte differentiation	1	5	3.19E−04	0.06	*AGT;IGF1;HAMP;GPER1;KAT2A;TBX3;MYLK3*
GO:0019216	Regulation of lipid metabolic process	4	3	3.60E−04	0.06	*FABP3;AGT;PDE3B;SQLE;IDI1;ABCD2;MVD;FDPS;GPER1;HMGCR;HMGCS1;STARD4*
GO:0006720	Isoprenoid metabolic process	1	6	4.61E−04	0.07	*IDI1;MVD;FDPS;HMGCR;CYP4V2;HMGCS1*
**Gene sets overrepresented among genes upregulated by *ZNF436* knockdown**						
GO:0045926	Negative regulation of growth	3	4	1.74E−05	0.01	*MT1F;MT1E;MT1M;DNAJB2;MT1A;MT2A;MT1G;DUSP10;MT1H;SEMA3A;SEMA7A;SOCS2*
GO:0055076	Transition metal ion homeostasis	2	5	4.49E−05	0.02	*MT1F;MT1E;MT1M;MT1A;MT2A;MT1G;ABCB6;MT1H;PICALM*
GO:0097485	Neuron projection guidance	3	4	2.64E−04	0.06	*NEXN;FEZ1;EZR;FAM129B;SEMA3A;CHN1;PALLD;GAB1;SEMA7A;VASP*
GO:0090066	Regulation of anatomical structure size	5	3	2.79E−04	*0.06*	*PREX1;EZR;ARPC1B;PFN2;LRRC8A;GCLM;ANXA7;ACTA2;SEMA3A;TMOD3;PICALM;ADRA1B;SEMA7A;TMOD2;VASP*
**Gene sets overrepresented among genes downregulated by NUP85 knockdown**						
GO:0043062	Extracellular structure organization	8	5	7.77E-16	6.60E-13	*TNC;SERPINE1;LOX;THBS1;CD34;PTX3;COL11A1;RGCC;FBLN5;FBN2;JAM2;DPT;ITGA11;COL1A1;CRISPLD2;CREB3L1;ENG;SDC1;NDNF;EFEMP2;NID1;LOXL3;AGTR1;BMP1;ERO1B;COL8A1;POSTN;FLRT2;RAMP2;SERPINH1;FBN1;PLOD3;CAPG;CD44;SMAD3;APBB2;PLOD1;COLGALT1*
GO:0007162	Negative regulation of cell adhesion	5	3	1.03E-05	1.09E-03	*TNC;SERPINE1;THBS1;RGCC;JAM2;COL1A1;IL20RB;MYADM;LOXL3;FAM107A;PLXNA2;CASP3;SNAI2;DUSP1;POSTN;ARG2;ANGPT1*
GO:0045444	Fat cell differentiation	5	3	6.43E-04	1.40E-02	*WNT5A;LEP;TGFB1I1;GPX1;CCND1;JDP2;SNAI2;FRZB;WNT5B;SMAD3;GPER1;CCDC3;ADRB1*
GO:0019216	Regulation of lipid metabolic process	8	2	0.006998547	7.82E-02	*PDGFRB;LEP;DKK3;LPGAT1;DHCR7;EGR1;FDPS;TXNRD1;IGFBP7;SNAI2;AGTR1;ADIPOR2;CNR1;GPER1;FHL2;CCDC3*

336 genes were differentially expressed between *NUP85* knockdown and control cells with FDR < 10%, of which only five were upregulated (Supplementary Table 7). Downregulated GO included “extracellular structure organization” and “regulation of lipid metabolic process” (Supplementary Table 8, Table 3). Expression of two genes reported to be involved in lipolysis were among *NUP85*-regulated genes [*NPR3* fold change (f.c.) 0.70, *ADRB1* f.c. 0.77] (Supplementary Table 7, Supplementary Fig. 3).

### Correlation between expression of GWAS genes controlling lipolysis and thermogenesis genes

Lipolysis is believed to be important, though not necessary, for brown and beige adipose thermogenesis^31^. To assess the relationship between novel lipolysis-regulated genes described herein and thermogenesis, we correlated the expression of the genes *ZNF436, STX16, NUP85, NID2, GGA3, GRB2* with thermogenesis markers^32^ in a separate cohort of obese women^33^. A variable number of thermogenesis genes were correlated to the lipolysis regulating genes (Supplementary Table 9). However, for the majority of these genes there was an inverse correlation between lipolysis and thermogenesis gene expression suggesting that the novel lipolysis genes described herein do not have a consistent effect on thermogenesis.

### Mendelian randomisation analysis

To assess relationship between BMI and WHRadjBMI and lipolysis we used Mendelian Randomisation analysis. We provide evidence of causal effects of spontaneous and stimulated lipolysis on BMI but not WHRadjBMI (Supplementary Table 10, Supplementary Figs. 4–5). These results should be interpreted with caution, as the selection of SNPs for mendelian randomisation analyses was non-standard, i.e., the SNPs were not GWAS significant. Whilst the results for spontaneous lipolysis are convincing, those for stimulated lipolysis are curious, as the MR Egger is null, despite the lack of horizontal pleiotropy and the NOME assumption being fulfilled (IGX2 = 1). In addition, it should be noted that the use of a smaller dataset for the WHRadjBMI MR analysis (GIANT consortium)^10^ compared to the SNP selection analysis (GIANT consortium plus UK Biobank)^1^ likely underestimates any causal effect of lipolysis on WHRadjBMI. We have also analysed directionally consistency for all BMI and WHRadjBMI GWAS SNPs for association with lipolysis in GENiAL. Results were significant for BMI but not WHRadjBMI (Supplementary Table 11).

## Discussion

**Figure 4:**
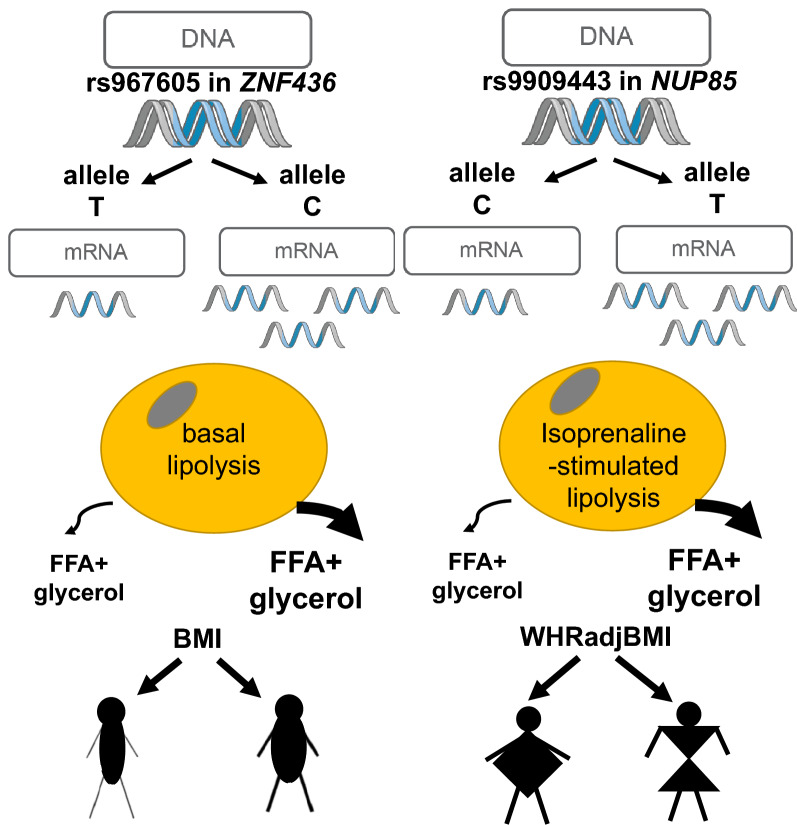
Summary figure demonstrating main findings for *ZNF436* and *NUP85*.

The findings reported here enhance our understanding of how genetic variants mediate their impact on the complex traits of fat storage and distribution. In the shared lipolysis-BMI or lipolysis-WHRadjBMI associated SNP panels we identified five and seven SNPs, respectively, displaying allele dependent gene expression for genes expressed in fat cells. Among these genes, we provided functional evidence supporting the hypothesis that transcriptional regulation of *ZNF436* and *NUP85* have intrinsic effects on lipolysis consistent with the associations in the clinical cohort and propose that these genes hereby are involved in the genetic regulation of BMI and WHRadjBMI (Fig. 4). Furthermore, candidate genes in one more BMI-locus (*STX17*) and two more WHRadjBMI loci (*NID2, GGA3* and *GRB2*) control lipolysis alone, or in conjunction with lipid storage, and may hereby be involved in body fat storage.

In the BMI-associated locus on chromosome 1 we showed that rs967605-C is associated with higher BMI, adipocyte spontaneous lipolysis, and *ZNF436* expression. Consistent with these associations found in clinical cohorts knock-down of *ZNF436* inhibited adipocyte lipolysis, whereas no impact on lipid accumulation was observed. ZNF436 is a member of the zinc finger transcription factor family and may act as a negative regulator on gene transcription mediated by MAPK signaling^26^. *ZNF436* mRNA is enriched in adipocytes, and in heart and brain as compared to cells from other organs^13,26^. *ZNF436* knock-down followed by transcriptome analysis revealed that ZNF436 acts as a regulator of many genes involved in lipid metabolic processes. However, ZNF436 did not control lipolysis via transcriptional regulation of hitherto described genes in the canonical human lipolysis process^30,34^. Importantly, the expression of a set of other genes reported to influence lipolysis or related processes, outside the canonical lipolysis pathway, was regulated by *ZNF436* knock-down (Supplementary Table 6, Table 4). These are therefore described in more detail below:

- *QRFP* encoding the neuropeptide 26RFa, which controls phosphorylation of HSL and PLIN1 and hereby inhibits lipolysis^35^.
- *PDE4A* encoding one subtype of phosphodiesterase 4, which inhibits lipolysis^36^.
- *ARHGAP26* which is involved in endocytosis and inhibits lipolysis^37^.
- *TRAF2* encoding TNF receptor-associated factor 2, which stimulates TNFA-induced lipolysis^38^.
- *TTC39B* encoding a scaffolding protein that promotes ubiquitination and degradation of liver X receptor, a transcription factor that in turn stimulates lipolysis. In addition, *TTC39B* antisense treatment protects against obesity in mice^39^.

**Table 4:** Genes differentially expressed between siZNF436 or siNUP85 and control cells.

Gene symbol	Probeset	Fold change	FDR (%)	Average (NegC)
**Genes differentially expressed between *siZNF436* and control cells**				
*QRFP*	TC0900011754.hg.1	1.42	9.4	9.57
*PDE4A*	TC1900006985.hg.1	1.36	9.4	7.49
*ARHGAP26*	TC0500008941.hg.1	1.42	6.3	9.01
*TRAF2*	TC0900009242.hg.1	0.67	6.3	6.76
*TTC39B*	TC0900012209.hg.1	1.27	9.4	8.78
**Genes differentially expressed between *siNUP85* and control cells**				
*ADRB1*	TC1000008968.hg.1	0.77	10.3	6.28
*BMPR2*	TC0200010489.hg.1	0.70	1.6	10.77
*EIF4EBP1*	TC0800007316.hg.1	0.66	1.6	10.71
*DDIT4*	TC1000007990.hg.1	0.52	0.0	11.23

On chromosome 9 rs10118701-G was associated with higher BMI, *STX17* expression, and lower stimulated lipolysis. Knock-down of *STX17* inhibited spontaneous lipolysis. We did not follow up these findings to determine whether the gene had an opposing inhibitory effect on stimulated as compared to spontaneous lipolysis consistent with the opposing genetic association in the clinical cohort. One example of a gene with such opposing effects is *PLIN1,* which suppresses spontaneous and facilitates stimulated lipolysis^40^. *STX17* encodes a key component of the autophagosome, which in turn has been functionally linked to lipolysis^41^.

In the WHRadjBMI-associated locus on chromosome 17 we showed that rs9909443-T is associated with higher stimulated lipolysis and *NUP85* gene expression, as well as lower WHRadjBMI. *NUP85* knock-down resulted in consistent inhibition of spontaneous and stimulated lipolysis without affecting lipids giving support to the hypothesis that *NUP85,* rather than other genes in this locus, is causally linked to lipolysis. Central fat accumulation has been associated with reduced stimulated lipolysis^42^, but the relationship between lipolysis and fat distribution is less straight forward for fat distribution than for BMI since there are depot specific differences in gene expression and fat cell metabolism between e.g. abdominal and femoral fat depots^43^. NUP85 encompasses an essential component of the nuclear pore complex important for nuclear transport but has also been reported to be involved in DNA replication and gene activation^44^. *NUP85* has also been reported to be involved in chemotaxis of monocytes via membrane clustering of *CCR2*^45^. However, CCR2 is expressed at extremely low levels in human adipocytes^46^ and this function of *NUP85* can therefore not be justified in in adipocytes. The inhibitory effect of *NUP85* knock-down on lipolysis was accompanied by downregulated expression of numerous genes. Potentially, downregulated expression of *ADRB1* could contribute to inhibition of stimulated lipolysis. Other *NUP85* regulated genes may mediate the effects of *NUP85* deficiency on lipolysis. *NUP85* regulated genes previously implicated in pathways of potential importance for lipolysis are listed below:

- *BMPR2* deficiency in adipocytes inhibits phosphorylation of the lipid-droplet-coating protein perilipin^47^ and hereby impairs stimulated lipolysis.
- *EIF4EBP1*. Simultaneous lack of *EIF4EBP1* and *EIF4EBP2* increases sensitivity to diet-induced obesity and insulin resistance in mice^48^ and is involved in the regulation of the lipid-droplet-coating protein ATGL^49^.
- *DDIT4* is a fasting-regulated p53 target gene. Forced expression of *DDIT4* augment lipolysis in adipocytes^50^. In summary, the herein reported regulation of lipolysis by *NUP85* describes a new role for this gene in adipose biology.

Further analysis of WHRadjBMI loci revealed that at the chromosomes 14 and 17 knock-down of *NID2*, *GRB2*, and *GGA3,* respectively, inhibited both glycerol release and lipid accumulation. The simultaneous effects of these genes on lipid accumulation and on glycerol release prevented us from drawing any conclusion as regards which metabolic processes link SNPs in these loci to body fat distribution. In the joint BMI-WHRadjBMI locus on chromosome 1 the inhibition of spontaneous lipolysis by *TBX15* knock-down was not consistent with the genetic data where the T allele was associated with high *TBX15* gene expression and low spontaneous lipolysis. It is therefore likely that other functions in adipose tissue linked to *TBX15* may mediate the impact of this locus of body fat storage^51^.

One limitation of the study is that no adjustment for multiple testing was used when selecting SNPs associated with lipolysis for functional follow up. The approach was justified by strong prior biological knowledge of the relationships between obesity and the cellular phenotype in question. We have tried to balance the risk of false-positives and negatives results by using a p-value threshold and combined this with a strong and clear biological rationale, that is the importance of lipolysis in the function of adipose tissue. Importantly, a causal relationship between lipolysis and BMI was supported by both Mendelian randomization analysis and overall enrichment of SNPs displaying directionally consistent effects on BMI and lipolysis. The lack of such evidence for WHRadjBMI may be related to limited power of the study and a more complex association between lipolysis in different fat depots and adipose distribution.

Another limitation is that we only looked for eQTL genes retrieved from the GTEx portal which only reports genetic effects on gene expression in tissue samples and not those limited to adipocytes. Thus, we may not have identified all BMI/WHRadjBMI-SNPs controlling gene function in adipocytes. Furthermore, among the genetic loci with genes controlling lipolysis in siRNA experiments, only rs146182298 (*NID2*) and rs1328757 (*PCK1*) were potential lead eQTL SNPs, i.e., they were among the SNPs most strongly associated with expression of the specific gene according to visual inspection in GTEx. A third limitation is that we only assessed lipolysis in abdominal SAT although other adipose depots might be more relevant^52^, but are even less accessible for sampling in adequate numbers. In addition, glycerol release was used to assess lipolysis. Glycerol represents an overall activation of the regulatory enzymes in the lipolysis cascade. Additional measurement of enzymatic activities and protein levels would be necessary to determine which steps in the lipolysis pathway is controlled by studied genes. Finally, gene knockdown may, due to strong effect on cell function, result in adaptive effects on metabolism not induced by specific functional genetic variants. To validate the effect of specific genes on lipolysis, knockdown experiments could be complemented with transient transection, or with e.g. single base substitution experiments. Such comprehensive experiments were, however, beyond the scope of this screening of genes in GWAS loci.

In summary, our study highlights that genetic susceptibility to central obesity might be associated with risk of impaired adipose lipolysis. We provided functional evidence supporting the notion that transcriptional regulation of *ZNF436,* and *NUP85* have intrinsic effects on lipolysis consistent with the associations in the clinical cohort and propose that these genes hereby are involved in the genetic regulation of BMI and WHRadjBMI (Fig. 4).

## Supplementary Information

Supplementary Tables.

Supplementary Figure S1.

Supplementary Figure S2.

Supplementary Figure S3.

Supplementary Figure S4.

Supplementary Figure S5.

Supplementary Legends.

## Data Availability

Summary statistics from the GWAS analysis of spontaneous and isoprenaline-stimulated lipolysis, experimental data and gene array data are available upon request (ingrid.dahlman@ki.se).
