# Supplementary figures and images for: Shared genetic loci for body fat storage and adipocyte lipolysis in humans
Source: Sci Rep. 2022 Mar 7;12:3666. doi: 10.1038/s41598-022-07291-4 (PMC8901764; doi:10.1038/s41598-022-07291-4)

## Slide 1
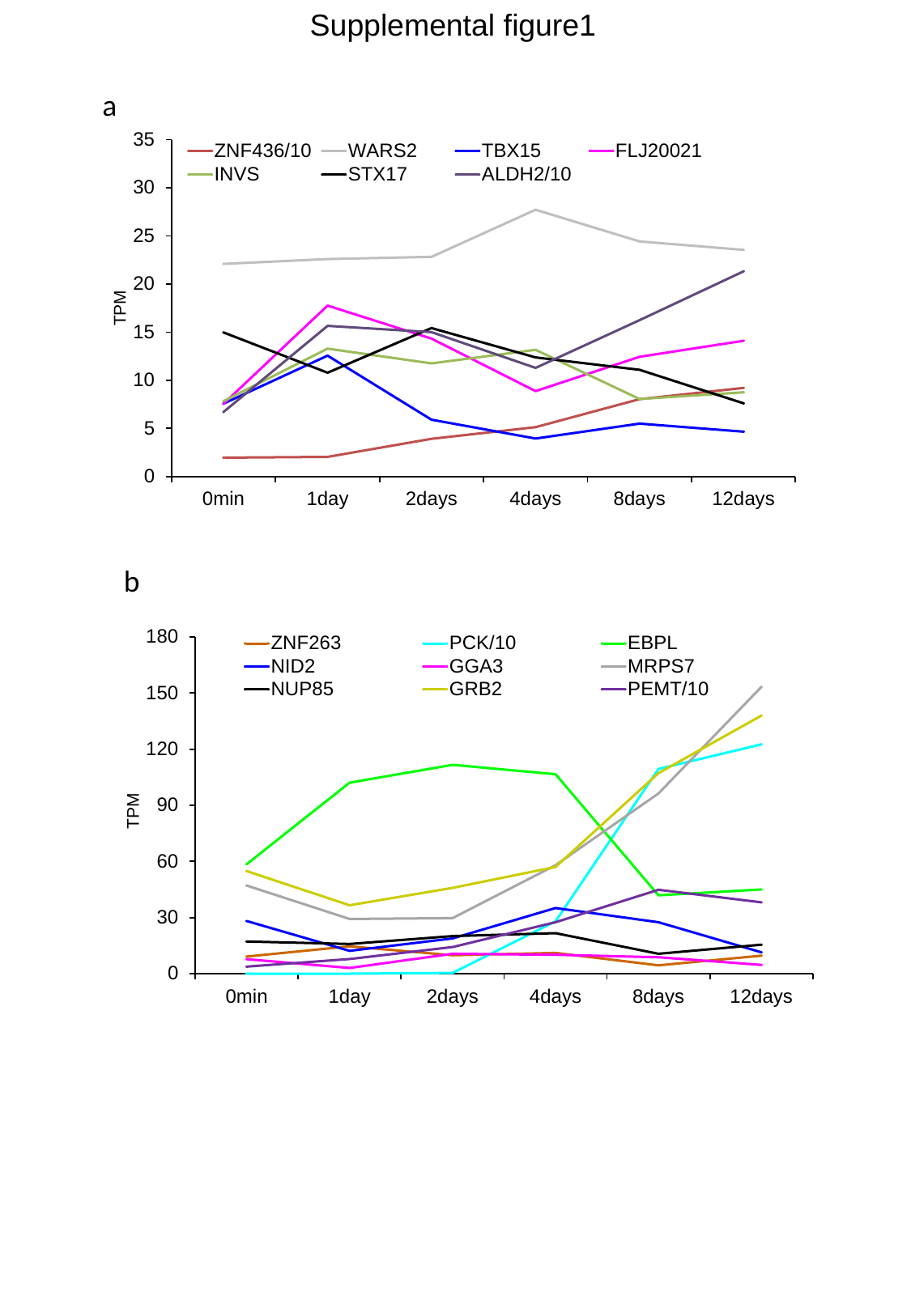

Supplemental figure1
a
b

Supplement: Supplementary file 2 — Supplementary Figure S1. [file 41598_2022_7291_MOESM2_ESM.pptx]

## Slide 1
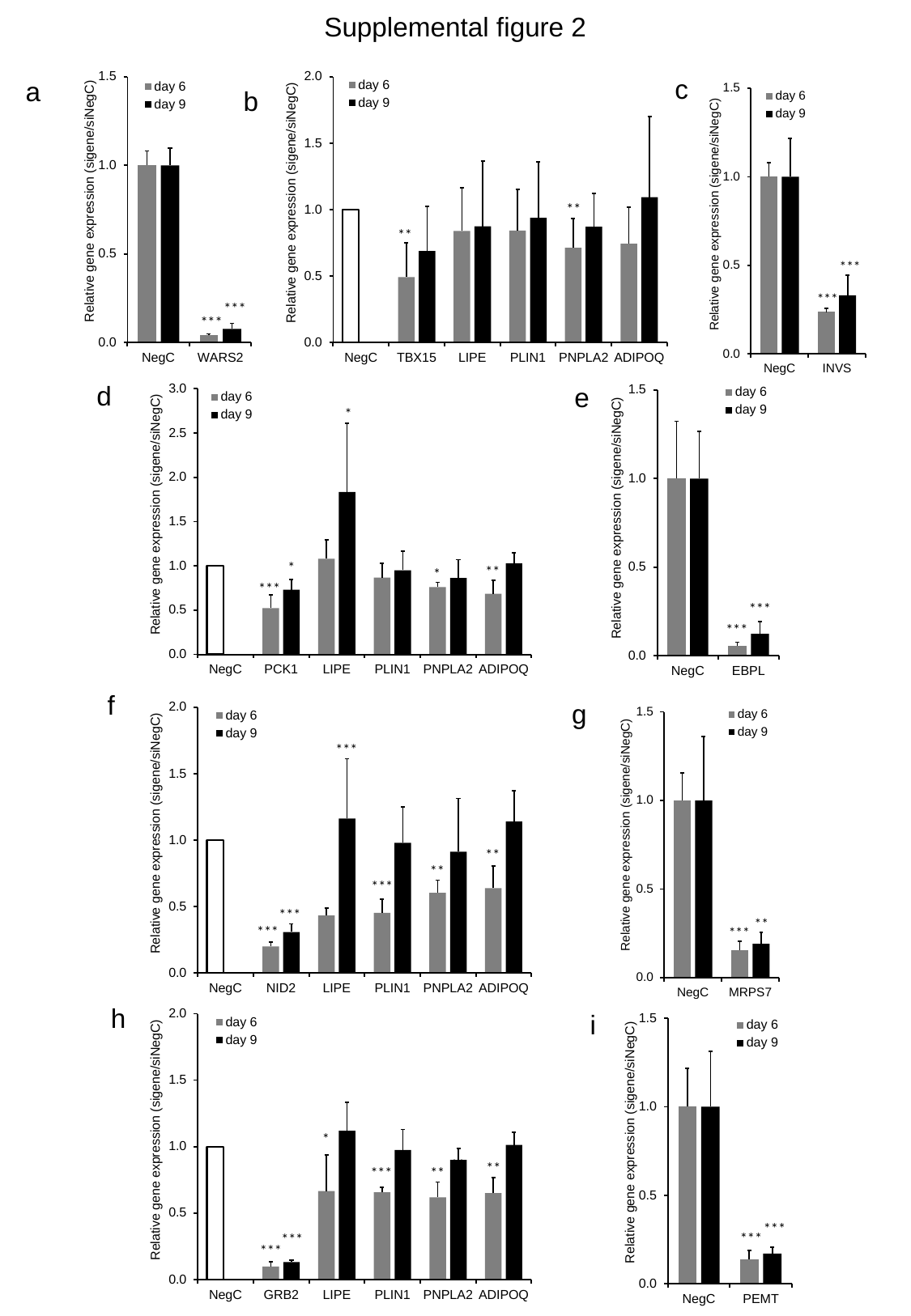

Supplemental figure 2
c
a
b
d
e
f
g
h
i

Supplement: Supplementary file 3 — Supplementary Figure S2. [file 41598_2022_7291_MOESM3_ESM.pptx]

## Slide 1
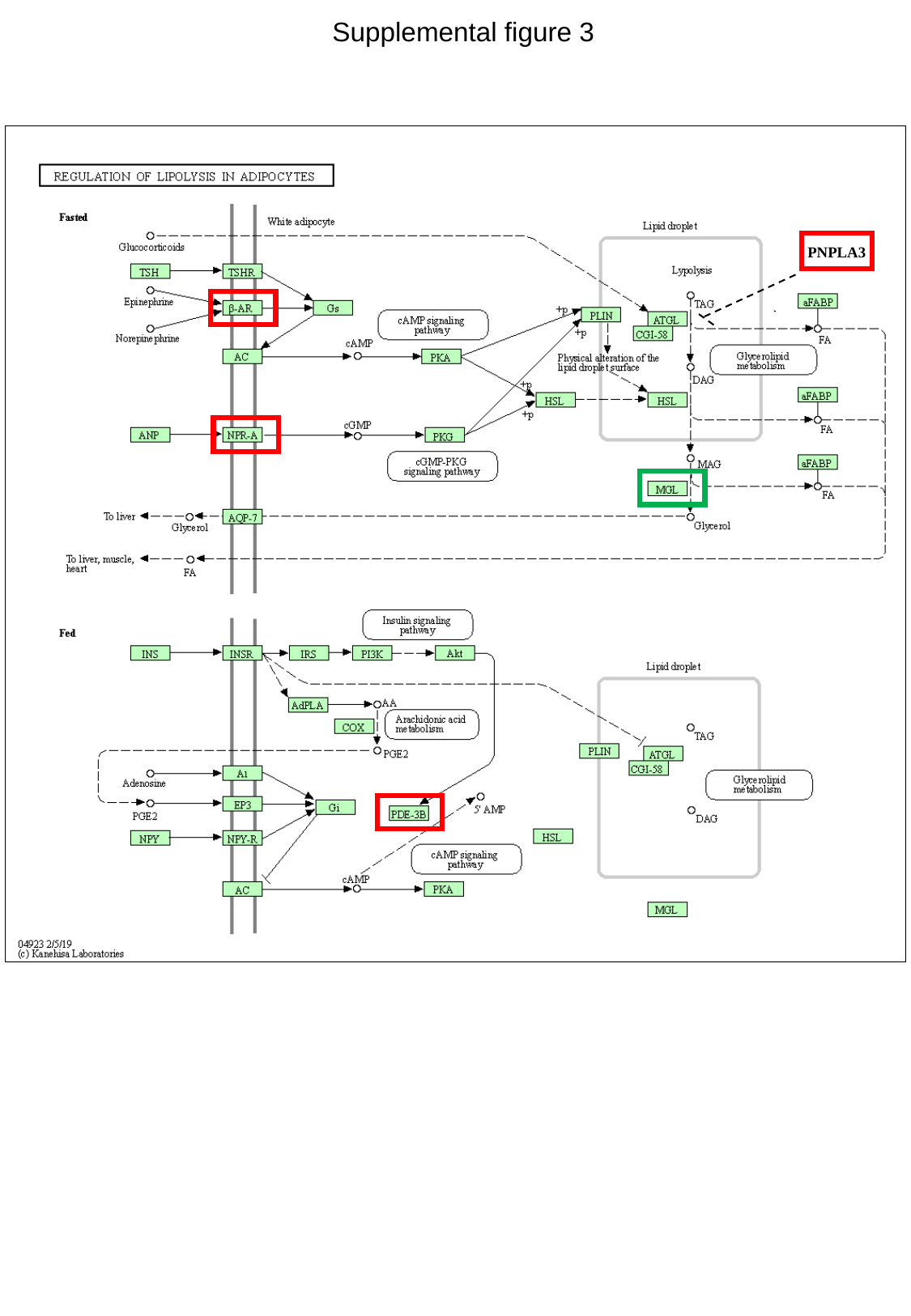

Supplemental figure 3
PNPLA3

Supplement: Supplementary file 4 — Supplementary Figure S3. [file 41598_2022_7291_MOESM4_ESM.pptx]

Supplemental figure 4

a

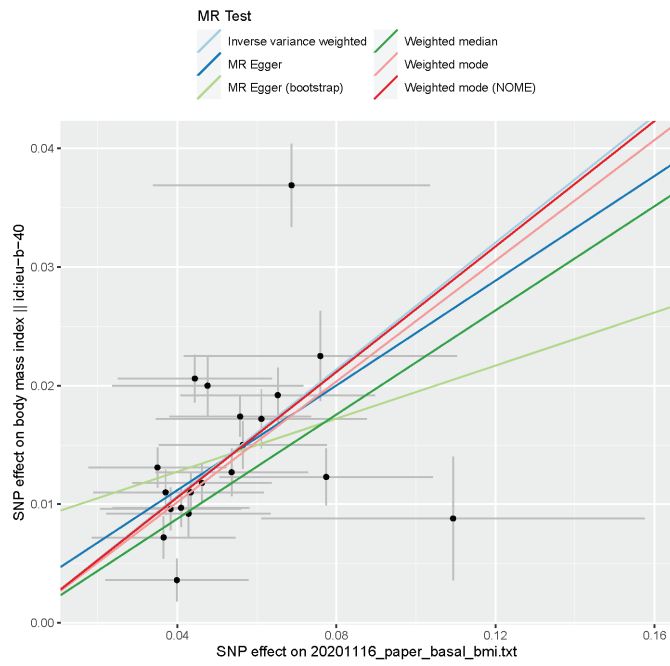

b

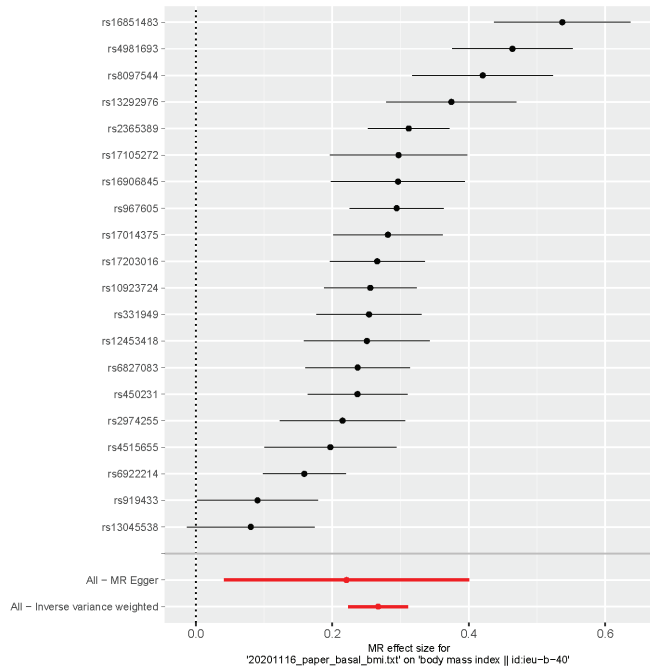

c

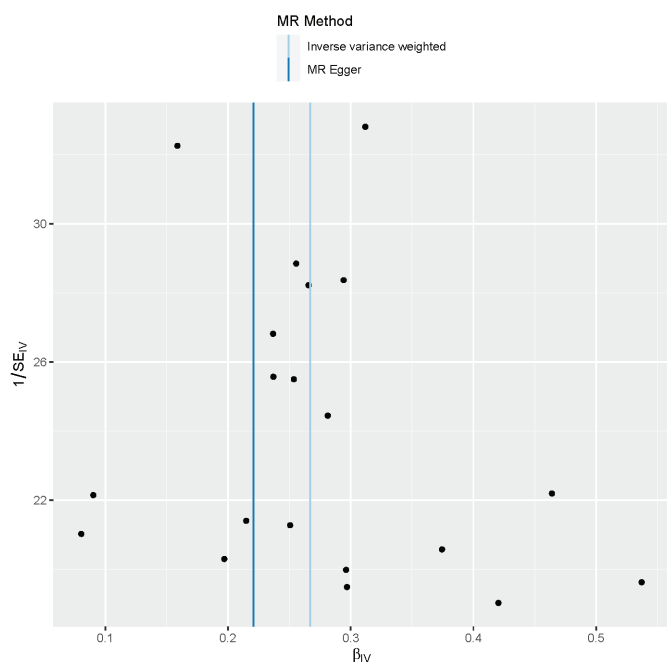

d

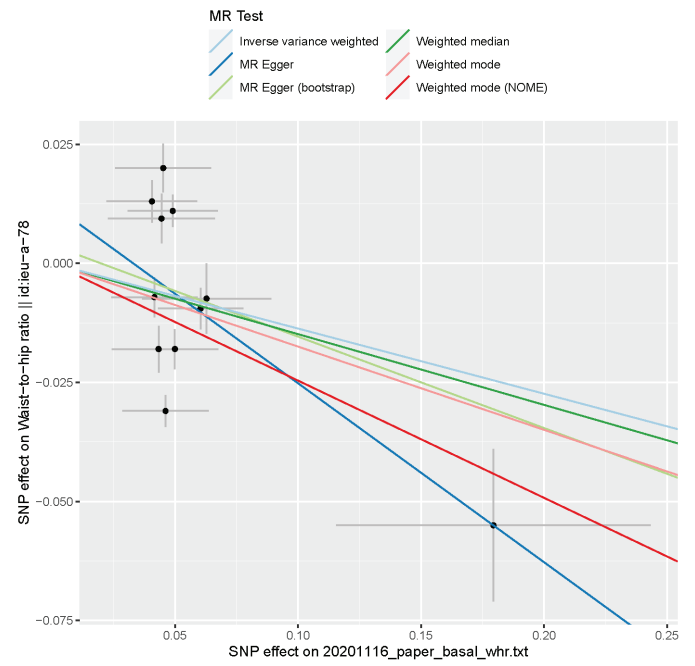

e

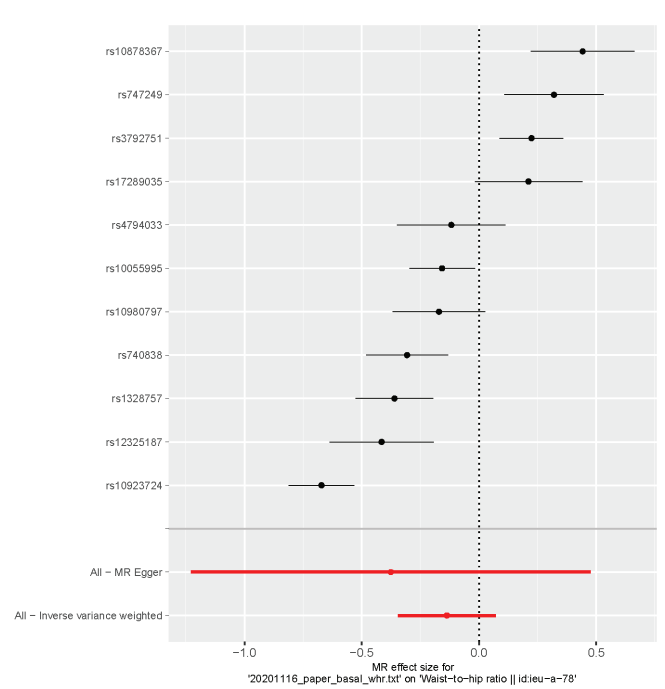

f

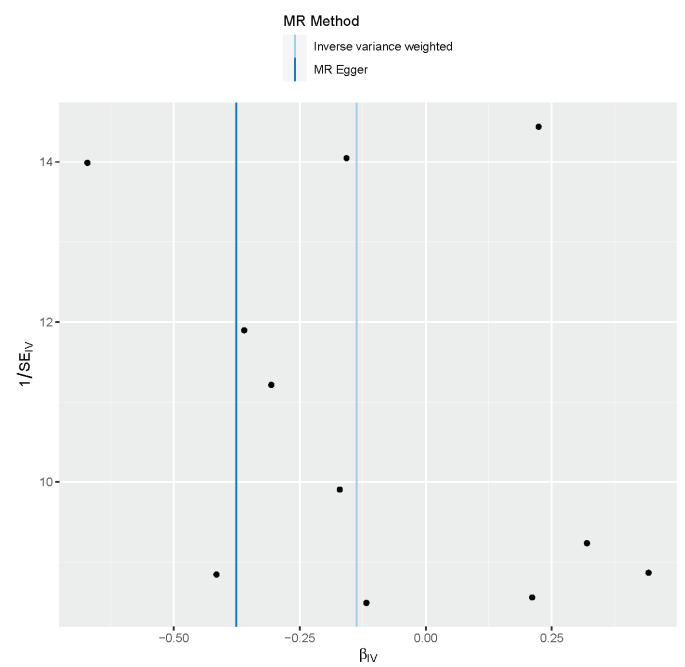

Supplement: Supplementary file 5 — Supplementary Figure S4. [file 41598_2022_7291_MOESM5_ESM.pdf]

Supplemental figure 5

a

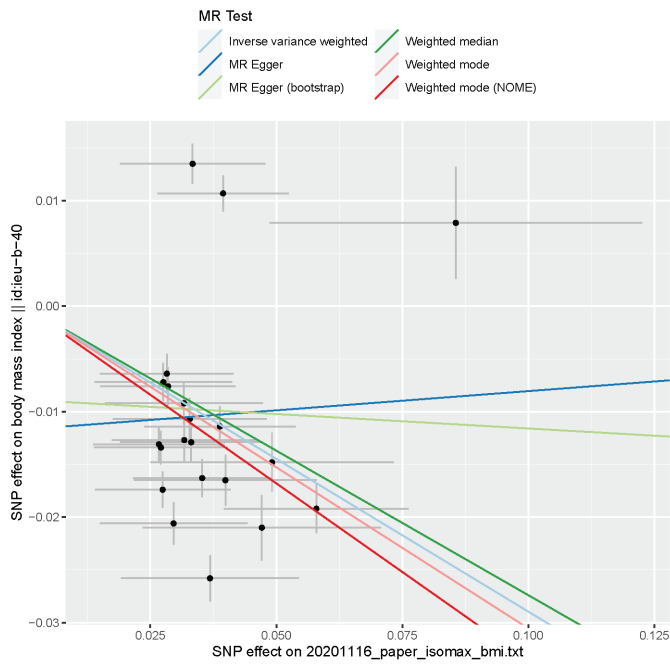

b

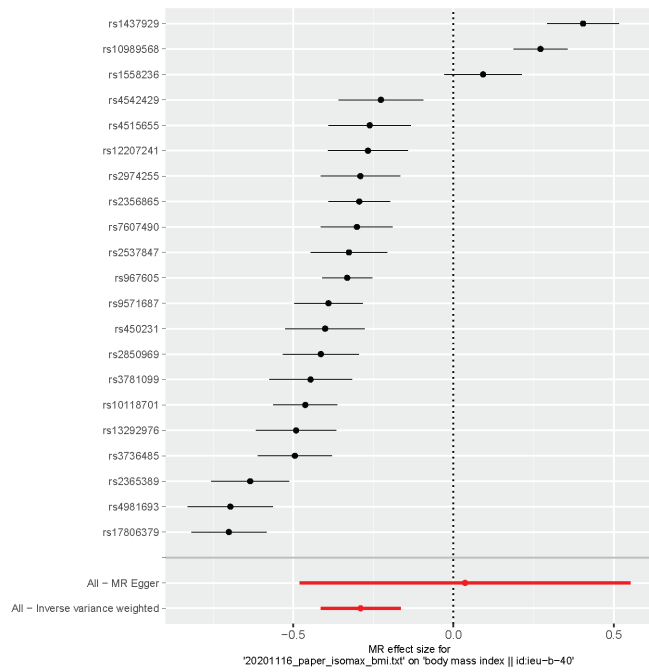

c

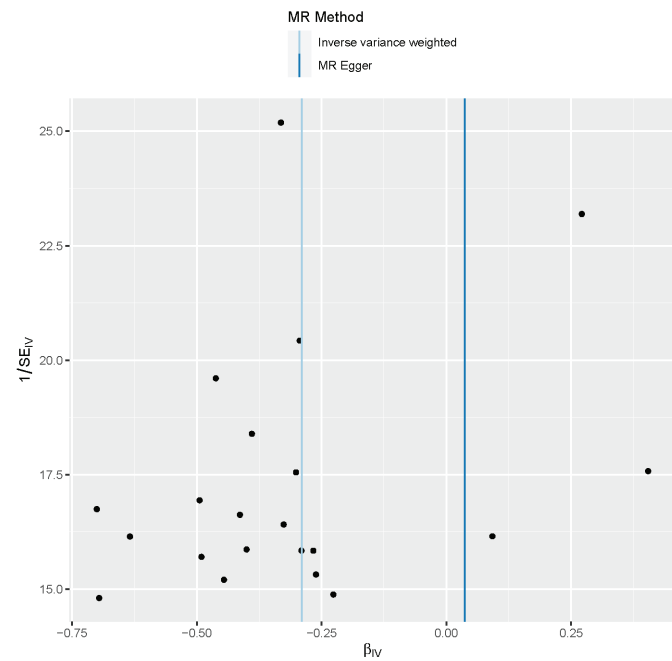

d

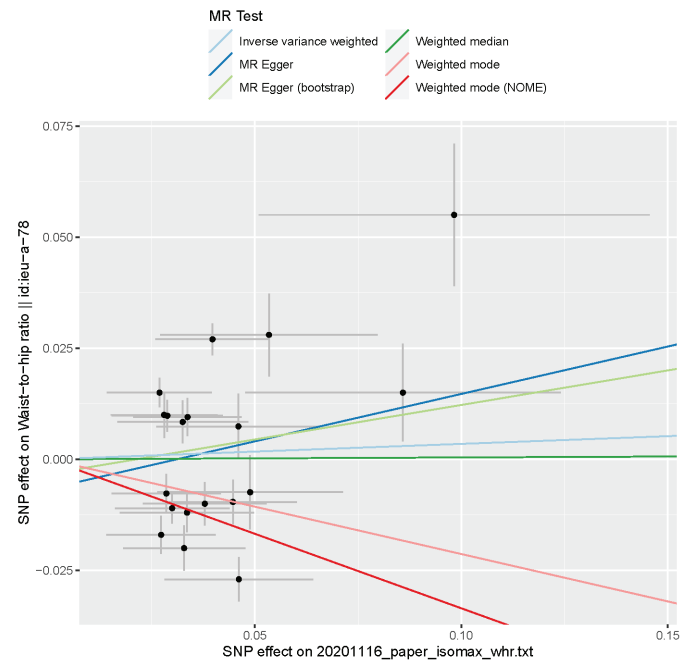

e

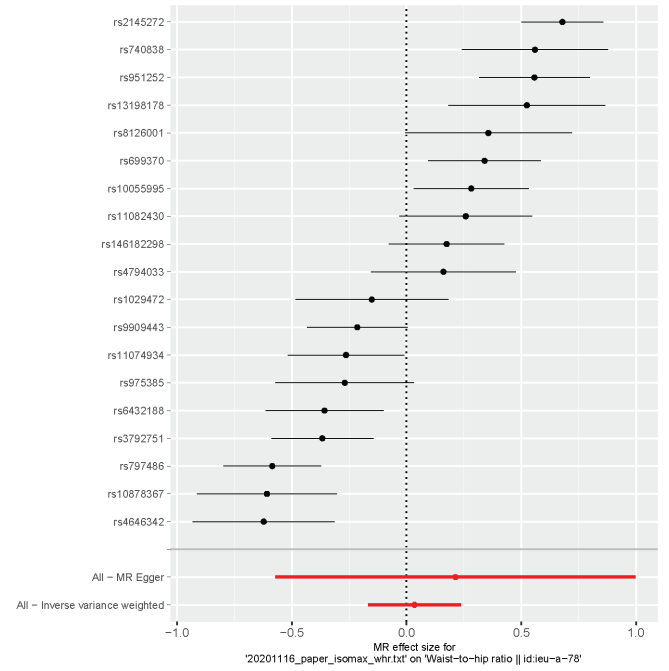

f

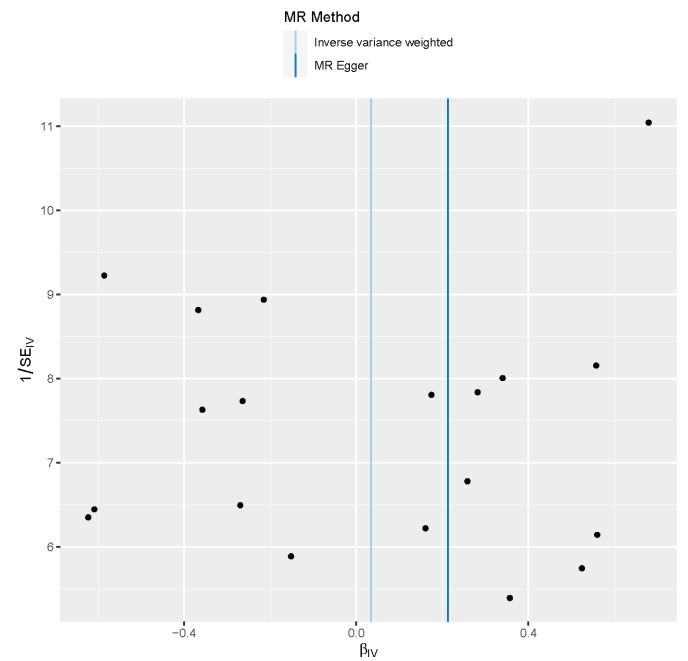

Supplement: Supplementary file 6 — Supplementary Figure S5. [file 41598_2022_7291_MOESM6_ESM.pdf]
