# Supplementary material for: Shared genetic loci for body fat storage and adipocyte lipolysis in humans
Source: Sci Rep. 2022 Mar 7;12:3666. doi: 10.1038/s41598-022-07291-4 (PMC8901764; doi:10.1038/s41598-022-07291-4)
Supplement: Supplementary file 7 — Supplementary Legends. [file 41598_2022_7291_MOESM7_ESM.docx]

**Supplementary figure legends**

**Supplemental figure 1. Expression of candidate genes during hASC differentiation *in vitro*.** Data retrieved From the FANTOM5 dataset (http://fantom.gsc.riken.jp/5/).

**a.** Expression of genes from BMI-loci.

**b.** Expression of genes from WHRadjBMI loci.

Expression of relatively highly expressed genes *ZNF436, PEMT* and *PCK1* genes were divided by 10 to facilitate visualization in the same graph. Tracks indicate normalized counts as TPM (tags per million).

**Supplemental figure 2. Effects of RNA interference-mediated knock-down of candidate genes in human adipose-derived stem cells** hASCs were transfected with control siRNA (siNegC) or indicated siRNAs targeting specific genes (*WARS2, TBX15*, *INVS, PCK1, EBPL, NID2, MRPS7, GRB2* and *PEMT*) and followed until differentiation day 6 and 9, upon which the expression of target genes and *LIPE, PLIN1, PNPLA2* and *ADIPOQ* (for *TBX15*, *PCK1*, *NID2*, *GRB2*) was monitored. Relative gene expression was normalized to the reference gene *18s*. Results were analyzed using t-test and presented as fold change ± SD relative to control siRNA (NegC) of corresponding time point (n=6, for *PCK1* n=8). ***P < 0.005, **P < 0.01, *P < 0.05.

**Supplemental figure 3.** Expression of *MGLL*, *PNPLA3* and *PDE3B* was regulated by *ZNF436* knockdown. Expression of *ADRB1* and *NPR3* was regulated by NUP85. Schematic figure Regulation of lipolysis in adipocytesis (hsa04923) is obtained from the KEGG database (22,23).

**Supplemental figure 4.** Comparison of Mendelian Randomisation (MR) methods, forest plots and funnel plots of SNPs included in the MR analyses of causality of spontaneous lipolysis on BMI (a-c) and WHRadjBMI (d-f).

**Supplemental figure 5.** Comparison of Mendelian Randomisation methods (MR), forest plots and funnel plots of SNPs included in the MR analyses of causality of stimulated lipolysis on BMI (a-c) and WHRadjBMI (d-f).
